# Food hygiene assessment in catering establishments in Hay Hassani district-Casablanca

**DOI:** 10.11604/pamj.2016.24.335.9171

**Published:** 2016-08-30

**Authors:** Nadia El Kadmiri, Halima Bakouri, Fatima Bassir, Saadia Barmaki, Laila Rachad, Sellama Nadifi, Omar El Kadmiri, Bouleghmane Amina

**Affiliations:** 1 Faculté Polydisciplinaire de Taroudant, Université IBN ZOHR, Taroudant, Morocco; 2 The Regional Laboratory of Epidemiology and Environmental Health, Casablanca, Morocco; 3 Laboratory of Medical Genetics and Molecular Pathology, Faculty of Medicine and Pharmacy, Hassan II University of Casablanca, Morocco; 4 IST Laboratory, Faculty of Science, Abdelmalek Essaâdi University, Tetouan, Morocco; 5 Medical Delegation, district of Hay Hassani Prefecture, Casablanca, Morocco

**Keywords:** Food hygiene, hygienic quality, catering establishments, Casablanca

## Abstract

Contaminated food is responsible for a significant amount of illnesses. In Morocco, it has become a worrying concern. Numerous awareness campaigns are conducted to warn the population against the risks of such scourge in ways that will prevent foodborne illness. Lawful commissions are in charge of examining and ensuring food safety in production and catering establishments, in addition to the assessment of food poisoning risks. The aim of this study is to evaluate the hygienic quality of food handling, preparation, and storage in catering establishments within Hay Hassani prefecture in Casablanca. During the period 2006-2012 a total of 1765 food samples were taken and examined for microbiological quality tests. As analyzed, 562 per 1765 samples are declared unhealthy for consumption. We note that some products were highly contaminated as compared to other products (p <0.001), specifically vegetable dishes, and meat dishes. In Hay Hassani district food is generally prepared and sold under unhygienic conditions, adequate corrective measures have been announced to improve hygienic practices.

## Introduction

Food hygiene is a scientific discipline describing a set of conditions and measures necessary to ensure the safety and suitability of food at all stages of the food chain. Contaminated food is responsible for abundant illnesses, whether in developed countries or developing ones, thus it generates significant health threats [1, 2]. In Morocco, it has become a worrying concern. Since 1980, reporting all cases of poisoning in Morocco became mandatory following the ministerial law No. 19 829DR / BF / MM. The collective food intoxication represents in Morocco 11% of poisoning food. Over 90% of collective foodborne infections are confirmed or probable of bacterial origin [3]. The Anti-Poison and Pharmacovigilance Center of Morocco (CAPM) had compiled 77,133 poisoning cases between 1980 and 2007 (apart from scorpion stings and envenomation) for 16 regions of the kingdom, with a fatality of 15.34‰ [4]. In this context, several commissions covering all the kingdom are in charge of inspecting and ensuring food hygiene in production and catering establishments to assess the risks of food poisoning. The aim of this study was to evaluate the hygienic quality of food in those facilities.

## Methods

**Sources of samples:** catering establishments in Hay Hassani prefecture in Casablanca, Morocco

**Selection of samples:** During the period 2006-2012, 53 inspections (1 inspection per establishment) were performed. A total of 1765 food samples was gathered and examined for microbiological quality. The preselection of catering establishments started by the most frequented to less visited by the population. All inspections are planned as part of an annual schedule, approximately 8 inspections per month. The dishes and products were selected, depending of their higher risks of contamination and their high frequency of consumption. The sampling number cannot exceed 16 samples per day. Foods are chosen from all varieties prepared and they will be served on the day of inspection. Samples were collected, placed in sterile vials, conserved in icebox and transported immediately to the laboratory for microbiological tests.

**Consent:** Our study doesn’t involve human subjects (including human material or human data). It involves just food samples.

**Laboratory tests:** Count of microorganisms invarious foods has been made according to the described method by Speck [5]. Ten grams or milliliters of each sample were transferred into 90ml (1%) peptone water. They were homogenized by grind in gland. An appropriate dilution was made for the microbiological study.

**Statistical analysis:** The data were analyzed using one way ANOVA, with the level of significance set at p <0.05. Statistical analyses were performed using the Graph PRISM V 6.01 software package.

## Results

**Figure 1:**
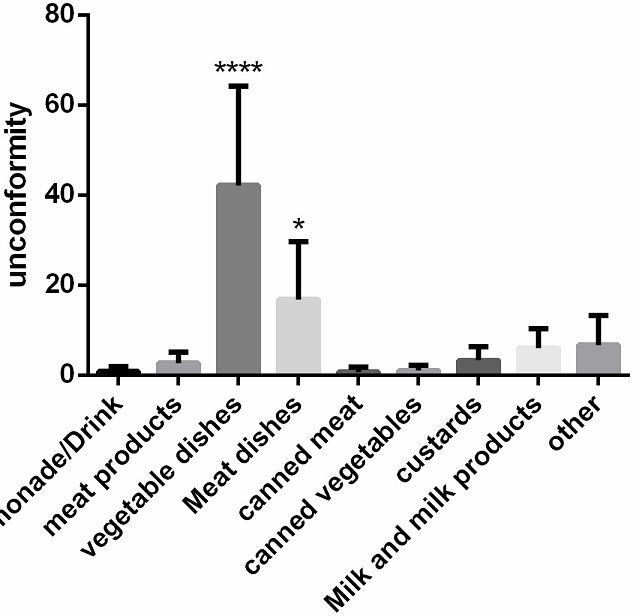
Analysis of the rate of discomfort food. Non significant difference of the rate over the seven years (P>0.05)

**Figure 2:**
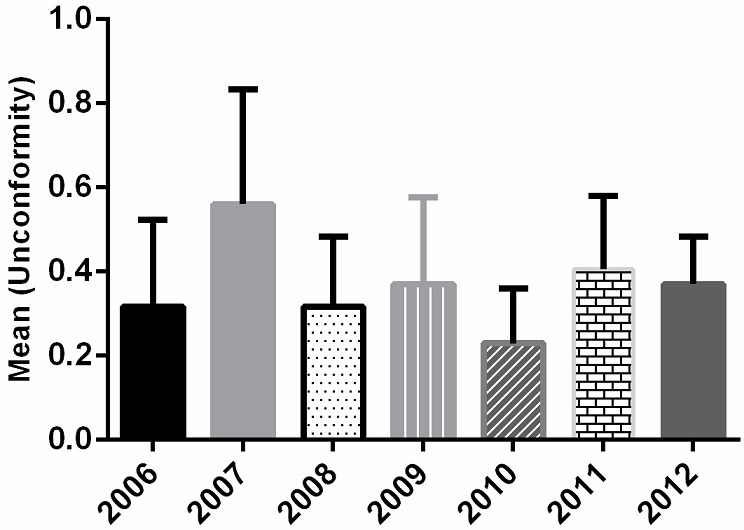
Analysis of discomfort rates foods according to their type. Vegetable dishes and hot dishes were higher contaminated as compared to other products (p <0.001)

Our inspections have revealed several anomalies regarding: hygiene of hands and body; storage of food and food preparation; separation of the hot and cold foods; freezing; food handling; hygiene of kitchen; packaging and evacuation of wastes. As analyzed, 562 per 1765 samples were declared un healthy for consumption. We note that some products were higher contaminated as compared to other products (p <0.001) (Figure 1), specifically products whose their preparation does not require cooking (vegetable dishes) or products susceptible to contamination and which after preparation are exposed for hours at ambient temperature (meat dishes). As observed these food products contained the highest incidence of coliforms as compared to other pathogens. During the seven years the mean of inconformity of the products did not change significantly (P>0.05) (Figure 2).

## Discussion

The level of instruction and training in good manufacturing practices, appropriate hygienic handling vary widely, and have been identified as major factors of contamination in this study and in different studies [6–8]. Unsuitable storage temperatures, mixing hot and cold foods, handling food with dirty utensils and without gloves expose all products to contamination and the consumer to risk of food poisoning [9]. An important factor that seems to be missing is training to learn hygienic practices. The role of inspections is very important to raise awareness, and to introduce corrective measures. Therefore, inspections must be strengthened to monitor the food quality in catering establishments. During our inspections, and after data analysis, a corrective measures [10] have been introduced to improve hygienic practices. The general measures mostly targeted food handlers who may act as source for infections: Food handlers should: have a medical file (medical examination, stool analysis and chest X-ray); wash their hands after defecation, during and after handling the food; use clean gloves and clean clothes; cover the hair; maintain a sanitary kitchen; avoid cross contamination between raw and cooked food; reduce time between food handling and service; maintain proper temperature of cooked food; protect prepared foods against rodent and insect contamination.

## Conclusion

The foods are generally prepared and sold under unhygienic conditions, with limited potential supply of clean water, and limited access to sanitation or garbage treatment facilities. Also, foods to be sold in streets have a higher risk of poisoning. In addition, foodborne illnesses are among the primary causes of death and constitute an economic burden in most countries. Exposure to the risk of poisoning could be avoided by the introduction of corrective measures through intensified inspections.

### What is known about this topic

- Foods are sometimes prepared and sold under unhygienic conditions;
- Food borne illnesses are a result of increasingly significant morbidity in all countries.

### What this study adds

- Our study highlights for the first time the microbiological quality of food and food hygiene during the period 2006- 2012 in Casablanca;
- Corrective measures have been introduced to improve hygienic practices.
