# Erratum: Food hygiene assessment in catering establishments in Hay Hassani district-Casablanca

**DOI:** 10.11604/pamj.2017.28.36.13254

**Published:** 2017-09-14

**Authors:** The Pan African Medical Journal

**Affiliations:** 1 The Pan African Medical Journal

**Keywords:** Erratum, hygiene, food, surveillance

## Abstract

This erratum corrects “Food hygiene assessment in catering establishments in Hay Hassani district-Casablanca.” The Pan African Medical Journal. 2016;24:335. doi:10.11604/pamj.2016.24.335.9171

## Erratum

This name of the author of this article [1] was mis-abbreviated on PubMed as “kadmiri NE”. The error was not present in both the PDF and HTML versions of the article. The author's name should be abbreviated “El Kadmiri N”.
