# Erratum: Food hygiene assessment in catering establishments in Hay Hassani district-Casablanca

**DOI:** 10.11604/pamj.2018.30.55.16066

**Published:** 2018-05-24

**Authors:** The Pan African Medical Journal

**Affiliations:** 1 The Pan African Medical Journal, Uganda, Kampala

**Keywords:** PAMJ, Erratum, food hygiene, hygienic quality, catering establishments, Casablanca

## Abstract

This erratum corrects article: “Food hygiene assessment in catering establishments in Hay Hassani district-Casablanca”. The Pan African Medical Journal. 2016;24:335. doi:10.11604/pamj.2016.24.335.9171.

## Erratum

The original version of this article [1] had the names of the author Nadia El Kadmiri wrongly abbreviated on pubmed KADMIRI NE instead of EL KADMIRI N.
